# Down-regulation of TGF- **β** 1 production restores immunogenicity in prostate cancer cells

**DOI:** 10.1054/bjoc.2000.1257

**Published:** 2000-07-24

**Authors:** E Matthews, T Yang, L Janulis, S Goodwin, S D Kundu, W J Karpus, C Lee

**Affiliations:** Department of Urology, Department of Pathology, Northwestern University Medical School, Chicago, IL 60611, USA

**Keywords:** TGF-β, expression, rat prostate cancer, immunogenicity, tumour incidence, host–tumour interaction

## Abstract

The objective of this study is to determine if a non-immunogenic Dunning’s rat prostate cancer cell line, MATLyLu, can become immunogenic by reducing the endogenous production of TGF-β1. An expression construct containing a DNA sequence in an antisense orientation to TGF-β1 (TGF-β1 antisense) was stably transfected into MATLyLu cells. Following transfection, cellular content of TGF-β1 reduced from 70 to10 pg per 2 × 10^4^cells and the rate of in vitro^3^H-thymidine incorporation increased 3–5-fold. After subcutaneous injection of tumour cells into syngeneic male hosts (Copenhagen rats), the tumour incidence was 100% (15/15) for the wild type MATLyLu cells and cells transfected with the control construct, but only 43% (9/21, *P*≤ 0.05) for cells transfected with TGF-β1 antisense. However, when cells were injected into immunodeficient hosts (athymic nude rats), the incidence of tumour development was 100% (10/10) for both the wild type MATLyLu cells and cells transfected with the control construct and 90% (9/10) for cells transfected with TGF-β1 antisense. These observations support the concept that MATLyLu cells are immunogenic, when the endogenous production of TGF-β1 is down-regulated. © 2000 Cancer Research Campaign


*British Journal of Cancer* (2000) **83**(4), 519–525
© 2000 Cancer Research Campaign
doi: 10.1054/ bjoc.2000.1257, available online at http://www.idealibrary.com on IDEAL®

### Down-regulation of TGF-β1 production restores immunogenicity in prostate cancer cells

**E Matthews[1], T Yang[1], L Janulis[1], S Goodwin[1], SD Kundu[1], WJ Karpus[2] and C Lee[1]**

[1]Department of Urology and [2]Department of Pathology, Northwestern University Medical School, Chicago, IL 60611, USA

**Summary** The objective of this study is to determine if a non-immunogenic Dunning's rat prostate cancer cell line, MATLyLu, can become immunogenic by reducing the endogenous production of TGF-β1. An expression construct containing a DNA sequence in an antisense orientation to TGF-β1 (TGF-β1 antisense) was stably transfected into MATLyLu cells. Following transfection, cellular content of TGF-β1 reduced from 70 to10 pg per $2 \times 10^4$ cells and the rate of in vitro $^3$H-thymidine incorporation increased 3–5-fold. After subcutaneous injection of tumour cells into syngeneic male hosts (Copenhagen rats), the tumour incidence was 100% (15/15) for the wild type MATLyLu cells and cells transfected with the control construct, but only 43% (9/21, $P \leq 0.05$) for cells transfected with TGF-β1 antisense. However, when cells were injected into immunodeficient hosts (athymic nude rats), the incidence of tumour development was 100% (10/10) for both the wild type MATLyLu cells and cells transfected with the control construct and 90% (9/10) for cells transfected with TGF-β1 antisense. These observations support the concept that MATLyLu cells are immunogenic, when the endogenous production of TGF-β1 is down-regulated. © 2000 Cancer Research Campaign

**Keywords:** TGF-β expression; rat prostate cancer; immunogenicity; tumour incidence; host–tumour interaction

Although TGF-β is inhibitory to many cancer cells in vitro (Laiho et al, 1990; Pietenpol et al, 1990), most tumours that express large quantities of TGF-β exhibit an aggressive phenotype (Barrack, 1997). On the other hand, tumours that have a reduced production of TGF-β show an attenuated growth pattern in vivo (Fakhrai et al, 1996). This difference of in vitro and in vivo responses of tumour cells to TGF-β is well known. What remains unclear is the impact by the host immune system on in vivo tumour growth due to an overproduction of TGF-β. The present report described the role of TGF-β production in host immune surveillance program against tumour growth.

Among the Dunning's rat prostate tumours, MATLyLu is the most aggressive line (Issacs et al, 1981; 1986). The MATLyLu system has been well characterized and is considered to be a good model for the late-stage, aggressive form of human prostate cancer (Smolev et al, 1977). These cells are androgen-independent and are highly invasive and metastatic. MATLyLu cells are known to be either non-immunogenic, or at best, weakly immunogenic (Shaw et al, 1987; Vieweg et al, 1994). They also produce a large amount of TGF-β1 (Steiner and Barrack, 1992; Barrack, 1997). Since TGF-β is a potent immunosuppressant (Letterio and Roberts, 1998), the lack of immunogenicity in these cells may be due, at least in part, to the large amount of endogenous production of TGF-β. MATLyLu cells are sensitive to TGF-β (Morton and Barrack, 1995). They demonstrate the typical paradoxical in vitro verses in vivo growth pattern in response to TGF-β1 (Barrack, 1997). Under culture conditions, TGF-β inhibits proliferation of MATLyLu cells. However, in animals, TGF-β1 enhances the tumorigenicity. This enhanced tumorigenicity may be the result of tumour–host interaction through a multitude of pathways.

In the present study, we demonstrate that the immunosuppressive effect of TGF-β plays a major role in MATLyLu tumorigenicity. The present results allow us to conclude that MATLyLu cells can be immunogenic when the endogenous production of TGF-β has been suppressed.

*Received 7 December 1999*
*Revised 22 March 2000*
*Accepted 26 March 2000*

*Correspondence to*: C Lee

### MATERIALS AND METHODS

### MATLyLu cells and culture conditions

MATLyLu cells were kindly provided by Dr John Isaacs of Johns Hopkins University at passage 53. Cells were routinely maintained in RPMI 1640 medium (Gibco Life Technologies, Gaithersburg, MD, USA) with 10% FBS (Summit, Ft. Collins, CO, USA), penicillin (100 units ml$^{-1}$), streptomycin (100 μg ml$^{-1}$) (Gibco), and 250 nM dexamethasone. All cells were cultured in a humidified atmosphere of 95% air and 5% $CO_2$ at 37°C. Cells under selective pressure were cultured as above with the addition of G418-sulphate (1000 μg ml$^{-1}$). Transfection of expression constructs to MATLyLu cells was performed at passage 71 and cells were used for the present study between passages 80–90.

### Construction of the TGF-β1 antisense expression vector

The pTARGET plasmid (Promega, Madison, WI, USA) is a mammalian expression vector containing the human cytomegalovirus immediate–early promoter region to allow constitutive expression of the cloned vector in host cells. This plasmid also contains a neomycin resistance gene, which was used for selection by G418-sulphate. The vector consisted of base pairs (bp) 433–1461 of the rat TGF-β1 cDNA in the reverse orientation and was inserted into the multiple cloning site of the pTARGET plasmid. Resulting constructs were digested with restriction enzymes and sequenced to confirm correct orientation, and designated as the TGF-β1 antisense construct. A construct with the empty vector (pTARGET plasmid alone) was prepared in the same manner, and designated as the control construct.

### Transfection and cloning

MATLyLu cells were transfected with the TGF-β1 antisense and control constructs with the Lipofectamine™ transfection system (Gibco) according to the manufacturer's recommended procedure. Briefly, cells were suspended in Opti-mem transfection media and were treated with a mixture of Lipofectamine™ and the above constructs (TGF-β1 antisense or control) for 18 h. Following transfection, cells were cultured with the maintenance medium and selected with G418-sulphate. Three days later, these cultures were transfected again and this procedure was repeated for a total of five times. Cloning was performed by limiting dilution into 96-well plates (Costar, Cambridge, MA, USA). Seven days after the initial plating, cells in a single colony were harvested and transferred into 24-well plates. At confluency, these cells were subsequently transferred into 25 $cm^2$ flasks and further expanded. Prior to injection of these cells into animals, the following tests were conducted.

### Verification of cloned MATLyLu cells by PCR

Confluent cultures were trypsinized and DNA was extracted via the Qiagen DNA preparation kit (Qiagen Inc, Chatsworth, CA, USA). DNA contents were measured by UV absorbence. An aliquot of 1.0 μg of genomic DNA was introduced into each PCR reaction vessel along with primers specific for a sequence within the vector. DNA sequences for the respective primers are listed below.

5′ primer-5′-GCACC AAAAT CAACG GGACT-3′ (bp 619–638)

3′ primer-5′-GAGAG AAAGG CAAAG TGGAT GTC-3′ (bp 995–1017)

An aliquot of 10 μl reaction buffer, containing 0.5 μg of Taq polymerase (ISC Bioexpress, Kaysville, UT, USA), 25 mM of dNTPS, 3 mM of $MgCl_2$, was added into each PCR reaction vessel and incubated for 35 cycles in a thermocycler (MJ Research, Watertown, MA, USA). Each cycle consisted of 95°C for 2 min, 55°C for 1 min and 72°C for 1.5 min. The PCR products were subjected to electrophoresis on a 0.8% agarose gel, stained with ethidium bromide, and were visualized under a UV lamp. The expected size (399 bp) of the PCR product was determined according to the DNA size reference, which was placed in the first and last lanes in the agarose gel for electrophoresis. Portions of the PCR products were also subjected to restriction digest and, then, to agarose electrophoresis to confirm the expected sequence.

### In vitro $^3$H-thymidine incorporation

$^3$H-thymidine incorporation was carried out to assess proliferative potential of each clone. Cells were seeded at 10 000 cells per well in 96-well plates and cultured in serum-free media. Twenty-four hours later, 1.0 μCi of $^3$H-thymidine (specific activity, 20–30 Ci $mmol^{-1}$, Amersham, Piscataway, NJ, USA) was added to each well. Cells were incubated for an additional 2 h and were subjected to two freeze–thaw cycles to detach cells from the wells. Suspended cells were transferred to a glass filter (Packard Instrument Company, Meriden, CT, USA) and were lysed with distilled water. Plates were allowed to dry for 2 h and an aliquot of 50 μl scintillation fluid was added. The amount of radioactivity was measured in a Scintillation Top Count Plate Reader (Packard Instrument Company). Results were expressed as counts per minute per well.

### Preparation of tumour and cell lysates for TGF-β1 determination

Levels of TGF-β1 in cell lysates and tumour tissues were determined by enzyme-linked immunosorbant assay (ELISA). Tumour tissues, weighing from 200–600 mg, were frozen with liquid nitrogen and ground to a powder. The solubilizing solution was added to each sample at a rate of 3 ml (phosphate buffer, 100 μg leupeptin) per 100 mg of tissue. Cell lysates were prepared by addition of 3 ml of the solubilizing solution to cell pellets ($10 \times 10^6$ cells). These samples were subjected to constant shaking at room temperature for 4 h and centrifuged for 15 min at 1500 ***g***. The clear supernatants were used for TGF-β1 determination. Briefly, an aliquot of 100 μl of the supernatant was activated with 100 μl 2.5 N HCl and 10 M Urea for 10 min. The activation step was necessary, as the amount of TGF-β1 in untreated samples was always negligible. Samples were neutralized with 100 μl of 2.7 M NaOH and 1.0 M HEPES prior to assay for TGF-β1. Samples were diluted 1:30 in calibrator diluent and subjected to ELISA for TGF-β1 (R&D, Minneapolis, MN, USA) according to the manufacturer's suggested procedure. Results were reported as pg TGF-β1 per 50 μg of tumour tissue or per $2 \times 10^4$ cells.

### Experimental animals

Copenhagen male rats (syngeneic hosts), Lewis male rats (allogeneic hosts), and athymic nude rats (immunodeficient hosts) were purchased from Harlan Industry (Indianapolis, IN, USA) at 60 days of age. Animals were kept in a temperature controlled room (23 ± 2°C) with tap water and normal rat chow provided ad lib. Experiments started at least 1 week after animals arrived at the facility. They were anaesthetized under methoxyflurane vapour (Schering-Plough, Union, NJ, USA). MATLyLu cells ($2 \times 10^5$ cells for syngeneic hosts and immunodeficient hosts, $I \times 10^6$ cells for allogeneic hosts) were suspended in 0.2 ml of serum-free culture medium and injected subcutaneously with a 25-guage needle into the left flank near the hind leg, while the animals were under anaesthesia. All procedures were approved by the Institutional Animal Care and Use Committee.

### Tumour measurement and tumour histology

Seven days following injection, rats were palpated twice weekly in order to monitor tumour development and tumour progression. When a tumour was palpated, the interval between tumour cell injection and tumour development was considered as the tumour-latent period. Tumour size was measured with calipers and tumour volume was determined by applying the formula (0.5236 (W + L) (W × L))/2, where W represents the width and L is the length (Janik et al, 1975). Animals were euthanized at 21–23 days by

*© 2000 Cancer Research Campaign*

decapitation while under anaesthesia with methoxyflurane vapour. Tumours were dissected and weighed. Tumours were cut into small pieces and were snap-frozen in liquid nitrogen for PCR analysis. A portion of each tumour was fixed in 10% neutral formalin. Tissues for histologic studies were embedded in paraffin, cut at 6.0 μm thick, and stained with haematoxylin and eosin. Photomicrographs of representative sections of the tumour tissues were taken with a camera mounted on a microscope (Olympus Model BH-2) as a hard-copy record.

### Statistical analysis

All numerical data were expressed as mean ± standard error of the mean (SEM). All in vitro experiments were repeated at least three times. Data were analysed using the analysis of variance test and Duncan's new multiple range test (Bender et al, 1982; Steele and Torrie, 1960). The Chi-square test was used to determine differences in tumour incidences in animals. A linear correlation was conducted to test the degree of association between the value of $^{3}$H-thymidine incorporation and the level of TGF-β1 production for different clones. A correlation coefficient (*r*) was calculated based on the analysis. A *P* value of less than 0.05 was considered as statistically significant (Steele and Torrie, 1960; Bender et al, 1982).

### RESULTS

### Verification of TGF-β1 antisense transfection in MATLyLu cells

A TGF-β1 antisense vector was created by inserting the full-length rat TGF-β1 cDNA into the multiple cloning site of the pTarget vector in the reverse orientation. MATLyLu cells were transfected with this construct, selected with G418-sulphate, and re-transfected in order to assure that high copy numbers of the vector were present. The repeated transfection appeared necessary, as MATLyLu cells produce high levels of TGF-β1. Early attempts using a single transfection episode were unsuccessful in reducing TGF-β1 production in transfected cells. It is likely that a great measure of antisense RNA must be present in the cytoplasm in order to suppress TGF-β1 production. Following serial dilution, clones were chosen. DNA from these cloned cells was isolated for PCR analysis. The positive detection of the expected 399 bp PCR product derived from transfected cell lines confirmed the presence of the transfected construct. Levels of TGF-β1 in cell lysates, as determined by ELISA, were significantly lower in clones transfected with the TGF-β1 antisense vector than in those of wild type MATLyLu cells and cells transfected with the control construct.

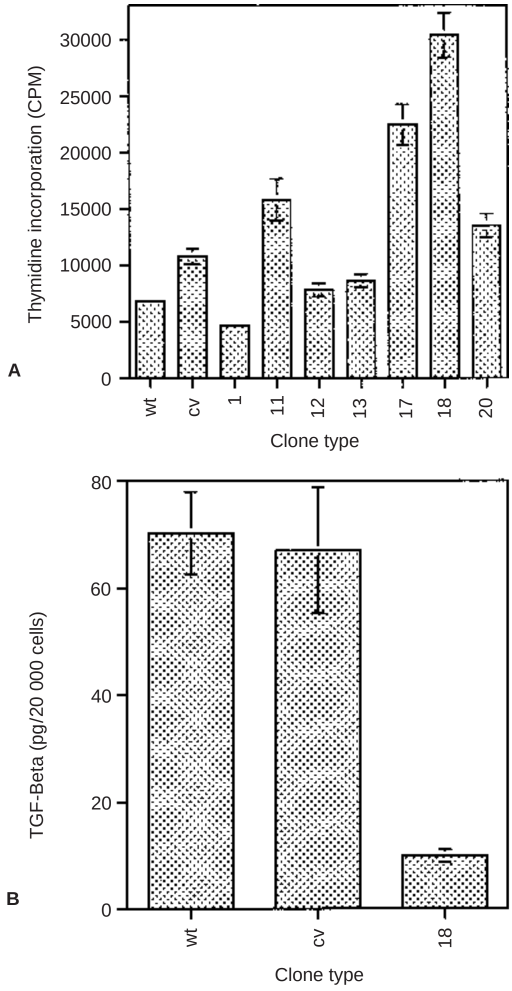

**Figure 1** The proliferation rates of wild type cells (WT), cells transfected with the control vector (CV), and different antisense clones in vitro (**A**), $^{3}$H-thymidine incorporation, and levels of TGF-β1 production (**B**) ELISA. Clone 18 (c18) was selected because it was the fastest growing and had the lowest level of TGF-β1 production of all of the antisense clones that were screened

The clone with the lowest level of TGF-β1 (clone 18) was chosen for further studies (Table 1 and Figure 1).

### $^{3}$H-Thymidine incorporation assay

$^{3}$H-thymidine incorporation in these cells was measured to determine proliferation rates of the various positive clones. There was a negative correlation between values of $^{3}$H-thymidine incorporation

**Table 1** Levels of TGF-β1 in cell lysates and $^{3}$H-thymidine incorporation rates of wild type MATLyLu cells, cells transfected with the control construct and cells transfected with the TGF-β1 antisense construct

| Cell type | TGF-β1 (pg per $2 \times 10^4$ cells) | $^{3}$H-Thymidine Incorporation (cpm per $1 \times 10^4$ cells per well) |
|---|---|---|
| Wild type MATLyLu cells | 70.3 ± 7.7 | 6804 ± 434 |
| Cells with control vector | 67.0 ± 11.8 | 10 774 ± 700 |
| Cells with antisense vector (Clone 18) | 9.9 ± 1.2[a] | 30 348 ± 2 866[a] |

All values are expressed as mean ± standard error of the mean. [a]The value is significantly different from the other values in the same column ($P < 0.05$)

**Table 2** Tumour incidence of MATLyLu cells inoculated subcutaneously into syngeneic, allogeneic, and immunodeficient hosts

| | Trial I | Trial II | Trial III | Overall |
|---|---|---|---|---|
| Syngeneic hosts: | | | | |
| Wild type MATLyLu cells | 5/5 (100%) | 5/5 (100%) | 5/5 (100%) | 15/15 (100%) |
| TGF-β1 antisense transfected cells | 3/5 (60%) | 4/11 (36%)[a] | 2/5 (40%) | 9/21 (43%)[a] |
| Control construct transfected cells | 5/5 (100%) | 5/5 (100%) | 5/5 (100%) | 15/15 (100%) |
| Allogeneic hosts: | | | | |
| Wild type MATLyLu cells | 4/4 (100%) | – | – | 4/4 (100%) |
| TGF-β1 antisense transfected cells | 0/5 (0%)[a] | – | – | 0/5 (0%)[a] |
| Control construct transfected cells | 4/5 (80%) | – | – | 4/5 (80%) |
| Immunodeficient hosts: | | | | |
| Wild type MATLyLu cells | 5/5 (100%) | 5/5 (100%) | – | 10/10 (100%) |
| TGF-β1 antisense transfected cells | 5/5 (100%) | 4/5 (80%) | – | 9/10 (90%) |
| Control construct transfected cells | 5/5 (100%) | 5/5 (100%) | – | 10/10 (100%) |

All values are expressed as mean ± standard error of the mean. [a] The value is significantly different ($P < 0.05$) from other values in the same group by the $\chi^2$-square test. In syngeneic hosts and immunodeficient hosts, a total of $2 \times 10^5$ cells were injected s.c. In allogeneic hosts, a total of $1 \times 10^6$ cells were injected s.c. Three separate trials were conducted for syngeneic hosts. One trial was conducted for the allogeneic hosts and two trials were conducted for immunodeficient hosts.

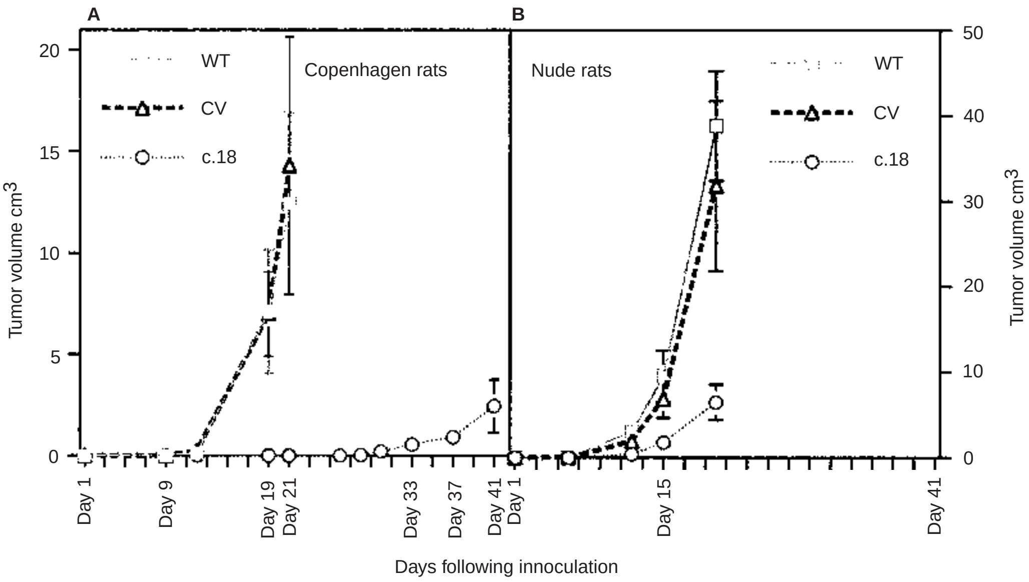

**Figure 2** Growth curves of tumours in Copenhagen rats (**A**) (syngeneic hosts) and in nude rats (**B**) (immunodeficient hosts). Tumour volumes were calculated according to the formula described in the text (Janik et al, 1975). Tumour volume in each group was calculated as mean ± SEM of all tumours. Tumours developed from the wild type MATLyLu cells (open squares) and cells transfected with the control vector (open triangles) were significantly bigger with a shorter latent period than those developed from the antisense vectors (open circles). Note that the scales in (**A**) and (**B**) are different, suggesting that tumours grow faster in immunodeficient hosts during the same interval. The vertical bars denote standard error of the mean.

and TGF-β1 levels in different clones ($r = 0.769$, $n = 7$, $P < 0.05$). Clone 18 has the highest level of $^3$H-thymidine incorporation, which was 3- to 5-fold higher than the proliferation rates for the wild type cells and cells transfected with the control construct (Table 1 and Figure 1). Therefore, this clone was chosen for further investigation.

### Tumour-latent period, incidence, and progression of MATLyLu cells in animals

The tumour-latent period is defined as the interval between tumour cell inoculation and detection of palpable tumour nodules. Table 2 shows, in male Copenhagen rats (syngeneic hosts), 100% of tumour development from wild type MATLyLu cells (15/15) and cells transfected with the control construct (15/15); but only 43% in animals injected with clone 18 cells (9/21). The average latent period for tumour development was 12 ± 0.54 days (mean±SEM) for the wild type MATLyLu cells and cells transfected with the control construct, for clone 18 cells, the average latent period was 25.2 ± 3.4 days ($P < 0.05$). Furthermore, tumours derived from clone 18 cells had an average weight (2.5 ± 1.3 g at day 41) significantly less than those derived from wild type MATLyLu cells (15.5 ± 4.3 g) and cells transfected with the control construct (12.6 ± 3.7 g) at day 21 (Figure 2A).

The experiment was repeated in male nude rats (immunodeficient hosts) and in Lewis rats (allogeneic hosts). As indicated in Table 2, tumour incidence of clone 18 cells grown in nude rats was 90% (9/10), which was not significantly different from those of wild type MATLyLu cells (10/10) or of cells transfected with the control construct (10/10). The latent period (8–11 days) of tumour

 © 2000 Cancer Research Campaign

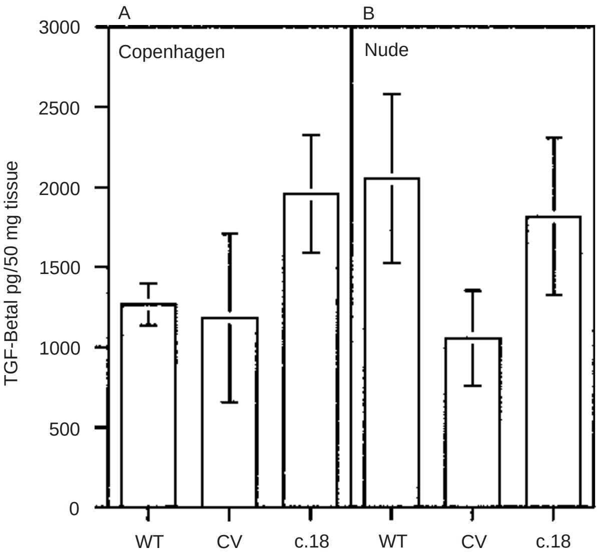

**Figure 3** Levels of TGF-β1 in tumours derived from Copenhagen rats (**A**) (syngeneic hosts) and from nude rats (**B**) (immunodeficient hosts). There were no statistical differences in TGF-β1. TGF-β1 levels (pg 50 $mg^{-1}$ tumour tissue) from wild type cells (WT), cells transfected with the control vector (CV), and cells transfected with the antisense vector (c18). Results are expressed as mean ± SEM for at least five tumours in each group. TGF-β1 was determined by ELISA as described in the text. The vertical bars denote standard error of the mean emergence was not significantly different, in contrast to the extended latent period observed in the syngeneic hosts. Furthermore, the tumour mass of clone 18 cells was significantly smaller ($P < 0.05$) from that of wild type cells or cells transfected with the control construct (Figure 2B). This observation suggests that for clone 18 cells, the host–immune responses as well as non-immune responses contributed to the reduced tumour growth in Copenhagen rats. Tumour incidence in Lewis rats (allogeneic hosts) was also studied in a small series. Results indicated that, in Lewis rats, there was a 100% tumour development for wild-type MATLyLu cells (4/4), 80% for cells transfected with the control construct (4/5) and 0% in animals injected with clone 18 cells (0/5) (Table 2).

### Characterization of MATLyLu tumours

Histologic features of tumours were similar to those reported in the literature (Isaacs et al, 1981, 1986). They contained highly undifferentiated tumour cells, resembling those of Gleason 5 anaplastic prostate adenocarcinoma. Mitotic figures were frequently seen. When tumour size was greater than 1.0 cm in diameter, central necrosis was evident. The periphery of the tumour was always surrounded by fibrous tissue derived from the host. In the present study, since tumours were harvested at a relatively early stage, the incidence of metastasis was not frequent. There were no apparent differences in morphologic features of tumours harvested from different treatment groups.

Results of PCR analysis for the presence of the expression vector indicated that clone 18 cells contained the expected 399 bp PCR product. Levels of TGF-β1 in the tumour lysates were determined by ELISA. As shown in Figure 3, in syngeneic as well as in athymic rats, tumours derived from the three different cell types showed comparable levels of TGF-β1. These results suggest that tumours derived from these cells all contain relatively high levels of TGF-β1 regardless of whether or not the original inoculum produced a low level of the growth factor.

### DISCUSSION

Results of the present study have demonstrated that MATLyLu cells, when the production of TGF-β1 is reduced, proliferate more rapidly under in vitro conditions but are less tumorigenic under in vivo conditions. The difference in growth behaviour of tumour cells under these two conditions is likely due to tumour–host interactions. It is widely accepted that wild type MATLyLu cells are non-immunogenic or, at best, weakly immunogenic (Shaw et al, 1987; Vieweg et al, 1994). Many experimental immunotherapy protocols have failed to cure the MATLyLu tumour (Vieweg et al, 1994). Results of the present study have demonstrated that the wild type MATLyLu cells produce high levels of TGF-β1 and are immuno-suppressive (Steiner and Barrack, 1992; Barrack, 1997). By simply reducing the production of the endogenous TGF-β1, MATLyLu cells showed a reduced tumour incidence in immune-competent hosts but not in immune-deficient hosts.

TGF-β is a potent immunosuppressant (Karpus et al, 1991; Leterio and Roberts, 1998). The notion that many tumour cells produce large quantities of TGF-β has been acknowledged (Gomella et al, 1992). Therefore, it is reasonable to propose that many tumour cells are immunosuppressive and that they are able to escape the host immune surveillance system. Basically, the present study observed the effect of TGF-β1 production by the tumour cells on three tumorigenic events: the incidence of tumour formation, the latent period, and the progression of established tumours. The first two events are clearly T-cell mediated, as there are significant differences in tumour incidence and the latent period between the syngeneic hosts and immune-deficient hosts. The third event may involve factors other than T-cell mediated immune system, as the impact of a reduced production of TGF-β1 on tumour progression is apparent in both syngeneic hosts and in immune-deficient hosts. These issues will be addressed below.

Xenograft growth of tumour cells in athymic hosts offers an opportunity to assess the behavior of tumour development under immune-compromised conditions. These animals are deficient in T cells but their natural killer (NK) cells remain functional. If tumours fail to develop in immune-competent hosts but grow in immunodeficient hosts, it is likely that the observed difference in tumour incidence is at least in past due to a functional T-cell immune system. In the present study, this is the case for tumour incidence and the latent period for tumour development, as the incidence has been significantly reduced and the latent period has been significant delayed in syngeneic hosts when compared with those events in the nude rats. Therefore, a reduction in TGF-β1 production in the tumour cells results in an escape from the T-cell mediated immunosuppression.

It is clear that the tumour–host interaction involves factors other than the T-cell immune system. These non-T cell factor may also play an important role in tumourigenicity. Nude rats, although lacking T cells, retain some immune function, as they still have NK cells, which may perform some tumour-suppressive functions. It is likely that the observed retardation in tumour growth in nude rats receiving clone 18 cells may result from the activation of NK

© 2000 Cancer Research Campaign cells due to the low levels of TGF-β1 produced by these cells. Differences in tumour growth may not completely attributed to the host immune system. It is apparent, from the present result, that the host immune system is one of many host factors that can be influenced by the production of TGF-β1 by tumour cells (Karpus et al, 1991; Leterio and Roberts, 1998).

In addition to immune suppressive function, TGF-β1 production by tumour cells can be responsible for many non-immune host factors, which cannot be ruled out at this stage. These are angiogenesis, stromal–epithelial interaction, expression of adhesion molecules, and production of extracellular matrix proteins (Battegay et al, 1990; Welch et al, 1990; Yang and Moses, 1990; Karpus et al, 1991; Barrack, 1997). These factors can promote tumour progression. In an environment of reduced TGF-β1 production, the in vivo tumour growth will be hampered. Results from studies with immunodeficient hosts indicate that tumours derived from the wild type MATLyLu cells and cells transfected with the control construct grow faster than those derived from the TGF-β1 antisense transfected cells do. These results indicate that the high levels of TGF-β1 produced by the wild type MATLyLu cells and by cells transfected with the control construct are able to stimulate a greater degree of tumour growth than that of the TGF-β1 antisense transfected MATLyLu cells. These factors may also contribute toward MATLyLu tumour growth.

An interesting finding in this study is that MATLyLu cells, when their TGF-β1 production is reduced, exhibit a low tumour incidence and a prolonged tumour-latent period in syngeneic hosts. When these tumour lysates were subjected to ELISA, however, the average level of TGF-β1 was not significantly different from that of the wild type tumours. Although results of PCR analysis indicate the presence of the antisense expression construct, the possibility could not be ruled out that some of the clone 18 cells might have lost their antisense constructs. We also acknowledge the possibility that a small fraction of the low TGF-β1-producing cells were never successfully transfected with the antisense vector. In either case, the consequence is the emergence of wild type cells in these tumours. The interesting aspect of this observation is that growth of these tumours appeared to be suppressed. A question has been raised of whether or not these TGF-β1-producing clone 18 cells would grow tumours as large as those derived from wild type MATLyLu cells, if they are left in Copenhagen rats for longer periods. We speculate that they would not grow as aggressively as the wild type tumours. This is because, when the low TGF-β1-producing clone 18 cells were injected into syngeneic hosts, they have been immunized and are capable of rejecting wild type tumour cells at least for a few months. This line of rationale is based on the report by Fakhrai et al (1996). These authors used the same method to transfect TGF-β2 antisense vectors into rat glioma cells and observed a similar reduction in tumourigenicity in syngeneic hosts. Subsequently, they re-challenged the wild type glioma cells to hosts who had previously rejected tumours. These re-challenged cells were also rejected, suggesting that, at that stage, host animals have been immunized to reject tumour cells even if they produce high levels of TGF-β.

In the present study, the acquisition of immunogenicity in low TGF-β1 producing MATLyLu cells was further substantiated by a lack of tumour growth in allogeneic hosts. In a preliminary study, tumours derived from wild type MATLyLu cells were eventually rejected by allogeneic hosts (Lewis rats) at 28 days following the initial tumour-cell inoculation. The growth of MATLyLu tumours in Lewis rats is a typical allogeneic transplantation rejection, which is characterized by an acute T-cell mediated reaction (Sherman and Chattopadhyay, 1993). However, during the first 14 days following inoculation of tumour cells, tumours grew from the wild type MATLyLu cells and cells transfected with the control construct, but not for TGF-β1 antisense transfected cells. This observation suggests that the high TGF-β1-producing tumours are endowed with a potent immunosuppressive shield, which protects them from the initial phase of allogeneic rejection.

In conclusion, the present observations have allowed us to conclude that MATLyLu cells, upon down-regulation of TGF-β1 production, become growth stimulated under in vitro conditions and growth inhibited under in vivo conditions. The in vivo growth inhibition is likely due to the presence of tumour–host interaction. The tumour–host interaction includes the host immune system and non-immune responses. Therefore, we propose that MATLyLu cells are actually immunogenic and that this immunogenic property has been masked by the endogenous production of a high level of TGF-β1. The high level of TGF-β1 in MATLyLu cells is a critical factor that renders these cells non-immunogenic. Reducing the endogenous production of TGF-β1 restores the immunogenicity in these cells. Our future studies will focus on cellular and humoral mechanisms that mediate such restoration of immunogenicity in MATLyLu cells. Understanding this mechanism may lead to the development of therapeutic programs based on lowering the level of TGF-β production in tumour cells.

### ACKNOWLEDGEMENTS

This research was supported in part by the US Army Medical Research and Materiel Command (PC97-410), Irwin and Sharon Grossinger Prostate Cancer Research Fund of Northwestern University Medical School, and the National Cancer Prevention Fund, Denver, Colorado, USA.

### REFERENCES

Barrack ER (1997) TGF-β in prostate cancer: A growth inhibitor that can enhance tumourigenicity. *Prostate* **31**: 61–70

Battegay EJ, Raines EW, Seifert RA, Bowen-Pope DF and Ross R (1990) TGF-β induces bimodal proliferation of connective tissue cells via complex control of an autocrine PDGF loop. *Cell* **63**: 515–524

Bender FE, Douglass LW and Dramer A (1982) *Statistical methods for food and agriculture*, pp. 887–107. The AVI Publishing Co Inc: Westport, CT, USA

Fakhrai H, Dorigo O, Shawler DL, Lin H, Mercola D, Black KL, Royston I and Sobol RE (1996) Eradication of established intracranial rat gliomas by transforming growth factor β antisense gene therapy. *Proc Natl Acad Sci USA* **93**: 2909–2914

Gomella LG, Sargent ER, Wade TP, Angland P, Lineham WM and Kasid BA (1992) Expression of transforming growth factor-α in normal human adult kidney and enhanced expression of transforming growth factor-α and -β1 in renal cell carcinoma. *Cancer Res* **49**: 6972–6975

Isaacs JT, Yu GW and Coffey DS (1981) The characterization of a newly identified, highly metastatic variety of Dunning R 3327 rat prostatic adenocarcinoma system: The MATLyLu tumour. *Invest Urol* **9**: 20–23

Isaacs JT, Isaacs WB, Feitz WF and Scheres J (1986) Establishment and characterization of seven Dunning rat prostatic cancer cell lines and their use in developing methods for predicting metastatic abilities of prostatic cancers. *Prostate* **9**: 261–281

Janik P, Braind P and Hartman NP (1975) The effects of estrogen-progesterone treatment on cell proliferation kinetics of hormone-dependent GR mouse mammary tumours. *Cancer Res* **35**: 3698–3704

Karpus WJ and Swanborg RH (1991) CD4+ supporessor cells inhibit the function of effector cells of experimental autoimmune encephalomyelitis through a mechanism involving transforming growth factor-β. *J Immunol* **146**: 1163–1168

 © 2000 Cancer Research Campaign

Laiho M, DeCaprio JA, Ludlow JW, Livinston DM and Massague J (1990) Growth inhibition by TGF-β linked to suppression of retinoblastoma protein phosphorylation. *Cell* **62**: 175–185

Letterio JJ and Roberts AB (1998) Regulation of immune responses by TGF-β. *Annu Rev Immunol* **16**: 137–161

Morton DM and Barrack ER (1995) Modulation of transforming growth factor β1 effects on prostate cancer cell proliferation by growth factors and extracellular matrix. *Cancer Res* **55**: 2596–2602

Pietenpol JA, Holt JT, Stein RW and Moses HL (1990) Transforming growth factor β1 suppression of c-*myc* gene transcription: Role in inhibition of keratinocyte proliferation. *Proc Natl Acad Sci USA* **87**: 3758–3762

Shaw MW, Rubenstein M, Dubin A, McKiel CF and Guinan PD (1987) Effect of cyclophosphamide on leukocytic subset distributions in rats carrying the Dunning R3327-MAT-Lylu prostatic adenocarcinoma. *Prostate* **11**: 117–125

Sherman LA and Chattopadhyay S (1993) The molecular basis of allorecognition. *Ann Rev Immunol* **11**: 385–402

Smolev JK, Heston WDW, Scott WW and Coffey DS (1977) Characterization of the Dunning R3327H prostatic adenocarcinoma: An appropriate animal model for prostate cancer. *Cancer Treat Rep* **61**: 273–287

Steele RGD and Torrie JH (1960) *Principles and procedures of statistics*. McGraw-Hill Book Co Inc: New York

Steiner MS and Barrack ER (1992) Transforming growth factor-β1 overproduction in prostate cancer: Effects on growth *in vivo* and *in vitro*. *Mol Endocrinol* **6**: 15–25

Vieweg J, Rosenthal FM, Bannerji R, Heston WDW, Fair WR, Gansbacher B and Gilboa E (1994) Immunotherapy of prostate cancer in the Dunning rat model: Use of cytokine gene modified tumour vaccines. *Cancer Res* **54**: 1760–1765

Welch DR, Fabra A and Nakajima M (1990) Transforming growth factor β stimulates mammary adenocarcinoma cell invasion and metastatic potential. *Proc Natl Acad Sci USA* **87**: 7678–7682

Yang EY and Moses HL (1990) Transforming growth factor-β1-induced changes on cell migration, proliferation, and angiogenesis in the chicken chorioallantoic membrane. *J Cell Biol* **111**: 731–741

*© 2000 Cancer Research Campaign*

## References

[PDF_00616] Barrack E. R. (1997). TGF beta in prostate cancer: a growth inhibitor that can enhance tumorigenicity.. Prostate.

[PDF_00618] Battegay E. J., Raines E. W., Seifert R. A., Bowen-Pope D. F., Ross R. (1990). TGF-beta induces bimodal proliferation of connective tissue cells via complex control of an autocrine PDGF loop.. Cell.

[PDF_00623] Fakhrai H., Dorigo O., Shawler D. L., Lin H., Mercola D., Black K. L., Royston I., Sobol R. E. (1996). Eradication of established intracranial rat gliomas by transforming growth factor beta antisense gene therapy.. Proc Natl Acad Sci U S A.

[PDF_00626] Gomella L. G., Sargent E. R., Wade T. P., Anglard P., Linehan W. M., Kasid A. (1989). Expression of transforming growth factor alpha in normal human adult kidney and enhanced expression of transforming growth factors alpha and beta 1 in renal cell carcinoma.. Cancer Res.

[PDF_00633] Isaacs J. T., Isaacs W. B., Feitz W. F., Scheres J. (1986). Establishment and characterization of seven Dunning rat prostatic cancer cell lines and their use in developing methods for predicting metastatic abilities of prostatic cancers.. Prostate.

[PDF_00630] Isaacs J. T., Yu G. W., Coffey D. S. (1981). The characterization of a newly identified, highly metastatic variety of Dunning R 3327 rat prostatic adenocarcinoma system: the MAT LyLu tumor.. Invest Urol.

[PDF_00637] Janik P., Briand P., Hartmann N. R. (1975). The effect of estrone-progesterone treatment on cell proliferation kinetics of hormone-dependent GR mouse mammary tumors.. Cancer Res.

[PDF_00640] Karpus W. J., Swanborg R. H. (1991). CD4+ suppressor cells inhibit the function of effector cells of experimental autoimmune encephalomyelitis through a mechanism involving transforming growth factor-beta.. J Immunol.

[PDF_00646] Laiho M., DeCaprio J. A., Ludlow J. W., Livingston D. M., Massagué J. (1990). Growth inhibition by TGF-beta linked to suppression of retinoblastoma protein phosphorylation.. Cell.

[PDF_00649] Letterio J. J., Roberts A. B. (1998). Regulation of immune responses by TGF-beta.. Annu Rev Immunol.

[PDF_00651] Morton D. M., Barrack E. R. (1995). Modulation of transforming growth factor beta 1 effects on prostate cancer cell proliferation by growth factors and extracellular matrix.. Cancer Res.

[PDF_00654] Pietenpol J. A., Holt J. T., Stein R. W., Moses H. L. (1990). Transforming growth factor beta 1 suppression of c-myc gene transcription: role in inhibition of keratinocyte proliferation.. Proc Natl Acad Sci U S A.

[PDF_00657] Shaw M. W., Rubenstein M., Dubin A., McKiel C. F., Guinan P. D. (1987). Effect of cyclophosphamide on leukocytic subset distributions in rats carrying the Dunning R3327-MAT-LyLu prostatic adenocarcinoma.. Prostate.

[PDF_00661] Sherman L. A., Chattopadhyay S. (1993). The molecular basis of allorecognition.. Annu Rev Immunol.

[PDF_00663] Smolev J. K., Heston W. D., Scott W. W., Coffey D. S. (1977). Characterization of the Dunning R3327H prostatic adenocarcinoma: an appropriate animal model for prostatic cancer.. Cancer Treat Rep.

[PDF_00668] Steiner M. S., Barrack E. R. (1992). Transforming growth factor-beta 1 overproduction in prostate cancer: effects on growth in vivo and in vitro.. Mol Endocrinol.

[PDF_00671] Vieweg J., Rosenthal F. M., Bannerji R., Heston W. D., Fair W. R., Gansbacher B., Gilboa E. (1994). Immunotherapy of prostate cancer in the Dunning rat model: use of cytokine gene modified tumor vaccines.. Cancer Res.

[PDF_00674] Welch D. R., Fabra A., Nakajima M. (1990). Transforming growth factor beta stimulates mammary adenocarcinoma cell invasion and metastatic potential.. Proc Natl Acad Sci U S A.

[PDF_00677] Yang E. Y., Moses H. L. (1990). Transforming growth factor beta 1-induced changes in cell migration, proliferation, and angiogenesis in the chicken chorioallantoic membrane.. J Cell Biol.

